# Dynamic influences on the neural encoding of social valence

**DOI:** 10.1038/s41583-022-00609-1

**Published:** 2022-07-12

**Authors:** Nancy Padilla-Coreano, Kay M. Tye, Moriel Zelikowsky

**Affiliations:** 1 Department of Neuroscience, College of Medicine, University of Florida, Gainesville, FL, USA.; 2 HHMI-Salk Institute for Biological Studies, La Jolla, CA, USA.; 3 Department of Neurobiology, School of Medicine, University of Utah, Salt Lake City, UT, USA.

## Abstract

Social signals can serve as potent emotional triggers with powerful impacts on processes from cognition to valence processing. How are social signals dynamically and flexibly associated with positive or negative valence? How do our past social experiences and present social standing shape our motivation to seek or avoid social contact? We discuss a model in which social attributes, social history, social memory, social rank and social isolation can flexibly influence valence assignment to social stimuli, termed here as ‘social valence’. We emphasize how the brain encodes each of these four factors and highlight the neural circuits and mechanisms that play a part in the perception of social attributes, social memory and social rank, as well as how these factors affect valence systems associated with social stimuli. We highlight the impact of social isolation, dissecting the neural and behavioural mechanisms that mediate the effects of acute versus prolonged periods of social isolation. Importantly, we discuss conceptual models that may account for the potential shift in valence of social stimuli from positive to negative as the period of isolation extends in time. Collectively, this Review identifies factors that control the formation and attribution of social valence — integrating diverse areas of research and emphasizing their unique contributions to the categorization of social stimuli as positive or negative.

Despite many social interactions being rewarding and capable of motivating instrumental behaviour from animals for access^1^, not all social interactions are positive. Indeed, sociability and valence have been proposed to be independent variables^2^. Here, we propose a model in which sociability and valence are linked by numerous factors — including social context, isolation history, social memory and social rank — that serve to influence the assignment of positive or negative valence to social stimuli, defined here as social valence.

What neural processes occur when an animal comes into contact with a social agent to rapidly evaluate the positive or negative valence associated with that social agent? Here, we explore the high-level computations that incorporate various contextual social factors that affect social valence, and the neural circuits and systems that implement them. We propose that social valence assignment depends on a combination of factors that influence the perceived valence of a social agent. In addition, we propose that these factors interact with the alignment between the self and other (social agent) to further influence valence. More specifically, the degree to which another social agent aligns with the self — that is, whether they share goals in a cooperation-like manner or have mutually exclusive goals in a competition-like manner — influences perception, valence assignment, motivation and action selection (FIG. 1). We define this phenomenon as social alignment, which can have a considerable impact on social valence.

Although social alignment is a primary factor that guides ongoing social valence assessment, many additional factors serve to influence social valence, particularly when there is a lack of familiarity with the social stimulus being assessed. When assigning valence to a novel social stimulus, we hypothesize that individuals will rely on the information they have — including social history, social attributes and their internal state, which is modulated by the preceding social environment. For example, in individuals with high familiarity, we propose that social attributes can influence assignment of valence, but that social memories, established ranks and established cooperative or competitive relationships will weigh more heavily on valence assignment than in interactions with unfamiliar agents. Similarly, we propose that factors impacting an animal’s social history (for example, experience of social isolation) can alter the hedonic value of social stimuli and, consequently, bidirectionally modulate the motivation to seek social contact^3,4^.

Social memory and recognition systems, as well as a catalogue of the social history of oneself and one’s relationships, are all necessary in order to represent whether a given relationship is cooperative or competitive. Although existing studies discuss social memory in a valence-independent manner, newer literature sheds light on the overlapping systems and circuits underlying social memory and valence. We review the neural systems that are necessary for social memory and recognition^5–8^, and how they contribute to the assignment of valence to a social stimulus. Furthermore, although social hierarchies have been described for a century, only recently have neuroscientists been studying their neural mechanisms^9,10^. We review this recent literature and how the dynamic nature of hierarchies relates to valence and could contribute to social valence assignment.

Social heuristics help guide the assignment of valence to a novel social stimulus, given its observable attributes. Across species, physical social attributes (how another individual looks, sounds and smells) provide crucial information that can modulate the valence assigned to that individual and, consequently, social motivation and the behavioural response to that individual (FIG. 2). For example, features that make an individual seem large and intimidating, or sick and weak, could decrease the drive to interact with that individual. We review the literature on how perception of social attributes guides social valence assignment.

In humans, perceived deficits in the objective quantity, or subjective quality, of social contact (‘loneliness’^11^) are correlated with deficits in mental^12^ and physical^13,14^ health, and shortened lifespan^15–19^. Perceived loneliness correlates with increased morbidity and mortality in cancer and cardiovascular disease^20^, and the severity of symptoms in response to viral immune challenges^21^ and of inflammatory responses^22^. Yet we are only beginning to uncover the neurobiological mechanisms that link deficits in social contact to the myriad of deleterious health consequences. Given the global isolation and distancing in recent years, one particularly timely question is how deficits in social contact change our brains and our behaviour. Prolonged social isolation can produce widespread and detrimental effects on the brain and behaviour across various species^23^, may result in dire evolutionary consequences^24^, produces territorial behaviour, aggression and social avoidance^25–30^, is considered torture^31^ and has even been used as a model for psychosis^27^. By contrast, acute periods of social isolation seem to have distinct effects on the brain and even opposing effects to those of extended social isolation on behaviour^3,32,33^. Does social isolation represent a singular internal state that lies on a continuum defined by time, changing its behavioural effects and biological underpinnings in a natural progression, or do acute and prolonged isolation represent separable internal states? Here, we synthesize research on the exciting intersection of sociality and valence, moving towards a framework for beginning to answer such questions.

## Social valence

Determining whether something is good or bad is one of the most important functions that the brain performs. Models of emotion posit that emotional states can be explained by how aversive or rewarding a stimulus is (valence) and how much arousal it evokes^34,35^. Here, we apply this general definition of valence to social stimuli. Similar to other stimuli, social stimuli are attributed as having a negative (unpleasant) or positive (pleasant) valence. This valence assignment is accompanied by observable changes in behaviour — approach towards a social stimulus of positive valence and avoidance of a negative-valence social stimulus. Many psychiatric disorders are characterized by dysregulated emotional processing and social behaviours, which could be driven by disruptions of valence encoding^36–39^. Furthermore, across psychiatric disorders, brain regions that encode both valence and social functions show aberrant activation during emotional processing^40^.

Appropriate valence assignment to social stimuli is necessary to seek and maintain healthy social lives and is vital for the survival of a social species. Unlike many other stimuli, social relationships are bidirectionally dynamic, involving flexible changes in behaviour of two active agents, both of which can affect the other. This makes the assignment of valence to social stimuli more complex than for other stimuli. Flexibility in social valence assignment is necessary to allow for changes in social motivation as a social relationship or the environment changes — seeking interactions when they are beneficial and avoiding them when they are not.

**Fig. 1 |:**
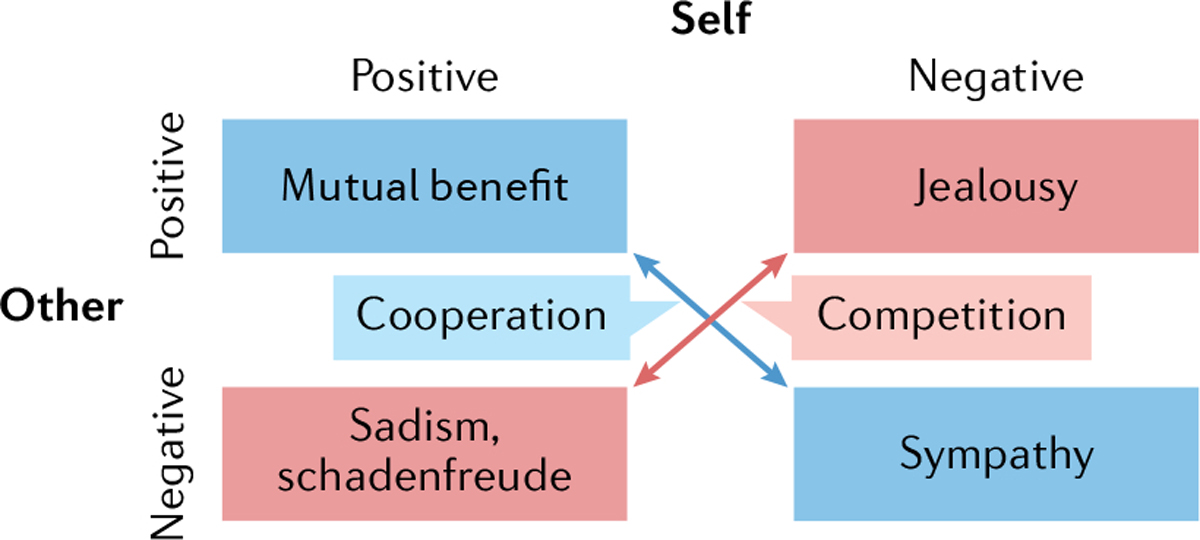
social alignment: a coarse parameterization of interactions between the self and other with positive or negative valence. Proposed model in which events can benefit the other and also be good for the self (resulting in mutual benefit); can be bad for the other and good for the self (jealousy, schadenfreude or sadism); can be good for the other and bad for the self (helping or altruism); or can be bad for both the self and the other, which could lead to sympathy. If an individual feels their fate is tied to another, then they will move along the axis of cooperation (events or stimuli that are good for the other are also good for the self, and punishments or threats to the other are bad for the self), and be motivated to help others (altruism)^250–252^. By contrast, if an individual perceives themselves to be in competition with the other, then they will move along the axis of competition (what is good for the other is bad for the self, and vice versa, as there are finite resources for which the self and the other are in competition)^253–257^.

The perceived valence of a social agent relies primarily on their social alignment: whether the relationship between oneself and the agent is competitive or cooperative (FIG. 1). The degree of opposition or alignment of two individuals’ goals is a primary parameter that defines the social relationship and can dictate social valence assignment during a given interaction. In addition, internal states, experience-based predictions and responses to a changing environment all influence the valence assigned to a social stimulus, implemented across multiple circuit motifs^41^. Computing social valence becomes highly complex, because it may incorporate the dynamic back and forth between two social agents. In addition, separate computations — each influenced by the relative rank, identity and history of previous interactions^3,4^ — may be made for each individual in a social interaction. Predictions of valence in terms of how others will behave and affect the self may be extrapolated based on individualized models for the theory of mind of each individual^42,43^.

Adding to this complexity, arousal can also influence the cognitive appraisal of an emotional or social stimulus, and individuals can experience emotional contagion in a way that is amplified by the arousal state^41,44–46^. Researchers have only just begun to unravel how the assignment of valence is algorithmically implemented into neural circuit motifs^41^. Emotions and their primitive variants are evolutionarily conserved, particularly among social mammals. Social contact due to group living can further affect social valence. Thus, communal dynamics, the evolutionary fitness of a species and the synchrony between behaving social agents are all major variables that can affect social valence (BOXES 1 and 2).

Although myriad factors contribute to social valence assignment and updating, we focus on the influence of social attributes, social rank and social isolation, as defined by a social homeostasis model (a model that proposes we have a preferred optimal ‘set point’ in terms of the quantity and quality of social contact, wherein social isolation or overcrowding may represent a challenge to the system)^3,4^. Although these key variables are universal factors that can affect valence assignment, variability in valence assignment across individuals can arise through differential weighting of these factors.

## Social attributes and their perception

The perception of physical social attributes requires multiple sensory systems, as social stimuli are multimodal. Here, we focus on auditory and olfactory cues, given the rich literary landscape about these, although we point the reader to emerging work in the still developing field of social touch^47–49^. We also briefly discuss some of the social visual perception literature that demonstrates valence encoding.

Importantly, these physical features do not intrinsically determine the social valence of a conspecific, because valence is assigned to a conspecific and, therefore, also depends on the state and social history of the subject assigning valence to the conspecific. The specific weighting and valuation of each feature may vary between individuals, and this variability may serve the evolutionary purpose of preserving diversity among a population. However, the physical attributes of a social stimulus represent a vivid and immediate source of information about many features of an animal, including its behaviour. Behaviours represent a dynamic weighted aggregate of information, whereas physical attributes are relatively static bits of information. Notably, auditory and visual information represent both static attributes (such as pitch of voice or size) and dynamic ones (such as vocalizations and gestures). How social stimuli are perceived can substantially affect social valence assignment.

### Perception of auditory social cues.

Conspecific vocalizations provide emotionally meaningful social information that informs behaviour. We learn to recognize voices as infants^50,51^, and our ability to perceive emotions from voices starts early in life, reported as early as 4 years old^52^. Voices can quickly convey universal emotion and arousal through screams, sobs or laughter^53^. Furthermore, voices can be used to identify gender^54^.

In the human brain, the superior temporal sulcus (STS) preferentially responds to human voices over other sounds^55^. In addition, the STS probably enables us to extract valence features about human voices, because it responds more strongly to emotional than non-emotional voices^56,57^ and is activated during voice gender perception^58^. The neighbouring superior temporal gyrus (STG) shows increased responses to emotional voices^57^. Notably, the STS and STG are involved in the multisensorial perception of both faces and voices^57^. Emotional vocalizations activate not only auditory regions but also the prefrontal cortex (PFC) and amygdala in humans^59^, suggesting that valence of social vocal cues could be assigned downstream of the auditory cortex. However, one study in humans showed that vocal emotions could be decoded based on the spatial patterns of blood oxygen level-dependent responses in auditory cortical regions, suggesting that valence information could be decoded during early perceptual processing of voices^60^. Other primates, such as marmosets and macaques, show vocalizations that are used to communicate and evoke responses in the PFC and amygdala^61–65^. Given the role of these regions in valence encoding^41^, these studies suggest that non-human primate calls may contain valence information.

Rodents emit ultrasonic vocalizations in the presence of conspecifics, and use these for social communication^66,67^; for example, to signal an affective state. In response to aversive stimuli, rats emit 22 kHz ultrasonic vocalizations^68,69^ that conspecifics use to learn about the valence of the stimuli despite not experiencing the stimuli directly^70^. In mice, ultrasonic vocalization emission is evoked by mating opportunities^71,72^ and to solicit maternal care^73,74^. Most research into rodent social vocalizations has focused on the neural circuits underlying their production, and thus little is known about their perception and the circuits that mediate their valence assignment.

### Perception of olfactory social cues.

Social odours carry crucial social information across species, such as kinship, health and sex^75–78^. Rodents, cattle and pigs can perceive chemosensory alarm signals from their conspecifics that signal potential danger^79–81^. Individuals with olfactory disorders, such as hyposmia and anosmia, report disruptions in their social life^82^, suggesting that olfaction has a social role in humans too.

In humans, smelling the sweat of students who were taking a final oral academic examination activated brain regions implicated in emotional processing such as the orbitofrontal cortex (OFC), insula and cingulate cortex, as well as the fusiform gyrus, which is activated by emotional cues of other modalities^83^. In another study, smelling chemosensory cues from individuals performing their first skydive produced strong activation of the left amygdala^84^. Intriguingly, in both studies, the participants did not report odour discrimination, suggesting that, despite the poor ability of humans to consciously perceive negative-valence signals in social odours, the human brain can perceive these signals and may alter behaviour without our awareness.

Rodents show an enriched ability to perceive social olfactory cues. Rodents can perceive predator and conspecific chemosensory cues, and these cues affect social behaviour^85,86^. Pheromones, non-volatile chemosensory cues, are crucial for rodent social communication. They mark territory, signal social dominance, mediate aggression and attract mates^87–91^. Neurons in the rodent accessory olfactory bulb (OB) respond to urine and saliva, sources of pheromones, to encode the sex and genetic strain of the social stimulus^92^. The medial amygdala (MeA) is involved in various innate social behaviours such as parenting, aggression and mating, and shows a high level of social experience-dependent plasticity^93–95^. Furthermore, in rodents the MeA is activated in response to pheromones to guide social behaviours^85^. Beyond the MeA, other brain regions are important for the perception of social odours. In female mice, a subpopulation of neurons in the medial preoptic area (mPOA) of the hypothalamus express the neuropeptide neurotensin, and respond to urine from males preferentially to appetitive smells^96^. Interestingly, optogenetic activation of this subpopulation is rewarding even in the absence of social stimuli, and evokes dopamine release in the nucleus accumbens (NAc), a structure involved in reward processing^96^, suggesting a direct overlap between positive-valence encoding and social-stimulus encoding in the hypothalamus. Rodents also show encoding of social olfactory stimuli in the PFC^97^. Furthermore, the rodent medial prefrontal cortex (mPFC) plays a part in valence encoding^98^. Whether the same subpopulations of mPFC neurons that encode valence of non-social stimuli also stably encode social stimuli and their valence remains an open question.

### Social cues of other modalities.

In addition to the modalities discussed above, social touch and visual perception of social stimuli are critical aspects of social interactions. Recent work suggests that the experience of social touch is subjective and involves the amygdala and other limbic structures^48,49^. For an in-depth review of social touch and its effects on emotional regulation, see REF.^48^. Visual perception also exerts a powerful influence on social valence; in particular, the perception of facial expressions in primates requires the face fusiform area^99^ and STS^100,101^, both of which encode valence information from faces^102,103^. Mice also show facial expressions in response to pain, positive or negative stimuli^104,105^, suggesting that rodent homologues of these regions could facilitate social communication in rodents as well. Overall, social attributes, signalled through various sensory modalities, can carry much inherent valence information.

Social scientists propose that social heuristics can provide simple intuitive rules to guide our social interactions via quick generalizations of social attributes from personal experience^106–109^. These social heuristics probably rely on the rapid perception of valence, on the basis of social sensory information.

## Social rank and valence assignment

Dominance hierarchies have long been described as a way in which social species organize group living^110^. Although the valence and social rank of a conspecific are unlikely to be independent variables, the relationship between them is unclear. It probably depends on the stability of the hierarchy and an individual’s social rank, given that these two factors influence the stress levels of an individual^111–113^. For example, dominant animals may find some social stimuli more stressful than do subordinates (for example, when the rank of the dominant animals is contested) and might find other stimuli less stressful than subordinates (for instance, when ranks are stable and resources are scarce). We propose a model in which the social context influences the perceived valence of social stimuli in a rank-dependent manner. Importantly, social valence can be modulated not only by perceived social rank but also by dominance expression. For example, a lower-ranking animal in a scarcity context might be forced to compete for resources and lose, making the valence assignment of a dominant individual negative.

In mice, an individual’s social rank correlates with neural activity differences in the PFC, amygdala, hypothalamic and brainstem nuclei^114–117^, and in macaques, social rank is associated with functional connectivity differences in many of these same regions^118^. These baseline rank-dependent differences probably modulate how the brain perceives the social rank of conspecifics. The complex relationship between an individual’s social rank and their perception of others’ social rank is not clear; however, a study in mice suggests that the neural response to social cues (urine samples from other male mice) is influenced by the social rank of the mouse perceiving the cue^116^. This study highlights the importance of measuring social rank as a variable of interest.

### Cortical encoding of social rank.

Most research on how the brain represents social rank comes from studies in primates. Social-rank perception might occur early in sensory processing, as, in humans, STG activity correlates with dominance ratings of facial expressions^119–121^. Also, participants asked to judge the social status of two unknown individuals showed increased functional MRI responses in the STS^121^. Given the role of the temporal cortex in perception of faces and valence in faces, we hypothesize that this STG and STS representation probably reflects processing of general facial and visual attributes linked to social standing.

The most robust representation of social rank is seen in the PFC, particularly the lateral PFC, both dorsal and ventral subregions. The human lateral PFC shows increased activation upon viewing high-ranking players or dominance-related postures^122,123^. In one of these studies^122^, destabilizing the hierarchy resulted in the mPFC becoming more active when viewing high-ranking individuals than low-ranking individuals.

This extra mPFC engagement in dynamic hierarchies is consistent with other studies showing that the mPFC is engaged by social-rank learning. In humans, functional MRI activity in the rostral mPFC correlated with social-rank perception, and stimulation of this region improves social-rank learning^124^. In mice, mPFC neural population activity is predictive of relative social rank during social competition^125^. Similarly, in mice, individual neurons in the anterior cingulate cortex (ACC) encode relative rank, reward size and success history during social competition^126^. Moreover, in humans, explicit judgement of social-status differences in a scene also engages mPFC activity^121^. However, one study suggested that the mPFC encodes social rank only when the participant is part of that hierarchy: when participants learned about two hierarchies (one including the participant, and the other not), their mPFC activity correlated with social-rank learning only for the hierarchy that included them^127^. This suggests that the mPFC helps track the social alignment between the self and others to guide valence assignment. Indeed, the mPFC represents self–other distinctions across species^125,128,129^, and functional MRI work in humans showed mPFC responses were clustered into those for self, familiar others and unfamiliar others^130^. Together, these results suggest that the mPFC tracks relevant information regarding the ‘self’ versus ‘other’ — including relative social-rank information — especially in dynamic situations.

In addition to the mPFC and lateral PFC, subpopulations of OFC neurons in monkeys respond differentially to familiar faces of dominant versus subordinate conspecifics^131^, suggesting that the OFC may also encode others’ social rank. The OFC also tracks outcomes for the self and for others during cooperative and competitive scenarios^132^. Both the mPFC and the OFC contain neurons that encode positive and negative valence^133,134^. Whether certain PFC cells encode both valence and social rank is not clear. However, considering how common mixed selectivity is in the PFC^135–137^, overlaps in valence and social-rank encoding probably exist.

### Subcortical encoding of social rank.

Compared with studies on cortical representation of social rank, the role of subcortical brain regions in encoding social-rank information has been less well studied. In macaques, single cells in the ventral striatum, a region important for reward processing, show changes in firing rate in response to dominant versus subordinate conspecifics’ faces, and overlap little with cells that respond to a liquid reward^138^. Thus, distinct neuronal subpopulations in the ventral striatum may encode valence and the rank of a conspecific.

When people are asked to learn the ranks of a group of men or a group of planets (as a non-social control), the activity of the anterior hippocampus (HPC) (analogous to the ventral hippocampus (vHPC) in rodents) and the amygdala correlated with the social rank recalled; however, the anterior HPC also tracked the non-social hierarchy^127^. Activity in the anterior HPC and amygdala also tracked social ranks in hierarchies including and not including the participants^127^. Furthermore, the amygdala and anterior HPC are coupled to the mPFC during updating of a hierarchy including the participant, but not during updating of a hierarchy that excluded the participant^127^. Amygdalar responses to high-ranking individuals were greater when participants were told a social hierarchy was unstable than when they were informed that social ranks were static and stable^122^. In macaques, the activity of the same amygdala cells that encode a rewarding stimulus predicts the social rank of conspecifics^139^, suggesting that the amygdala, unlike the striatum, uses valence-coding systems to encode social rank of conspecifics as well. Whether social-valence encoding guides social motivation in a rank-dependent manner remains unknown.

### Circuits modulating hierarchy updating.

Several recent optogenetic studies in mice are shedding light on the specific circuits that carry social rank information and affect social-dominance behaviour. Nonspecific stimulation of the dorsal mPFC increases social-dominance behaviour and, often, subsequent social rank in male mice^114^. Two studies have implicated a thalamo-cortical circuit in social dominance. One showed that projections from the mediodorsal thalamus (MDT) to the dorsal mPFC undergo plasticity with winning that reinforces social-dominance behaviour, and that optogenetic stimulation of the MDT–dorsal mPFC circuit is sufficient to induce winning and increase social rank^115^. The other study showed that lesioning the MDT slowed the formation of a hierarchy, and that bidirectional manipulation of MDT modulated winning behaviour in a social-dominance task^140^. The same study showed that parvalbumin-expressing interneurons in the ACC receive direct projections from the MDT and modulate dominance behaviour. Altogether, these studies show that this thalamus–PFC pathway is important for establishing and maintaining social hierarchies. MDT inputs to the mPFC are probably necessary for the prefrontal representation of relative social rank observed in other studies across species^124,125,127^. Inputs from the basolateral amygdala (BLA) and vHPC to the mPFC are probably also necessary for social-rank encoding and may modulate social dominance behaviour, given the functional connectivity observed during hierarchy updating^127^, the role of the vHPC in social memory^141^ and the role of the BLA in valence associative learning^142^.

As the amygdala, HPC and mPFC signal social rank and are functionally connected, we hypothesize that social-rank information is transmitted from the mPFC and vHPC to the BLA, where it is integrated with valence information, and that it returns from the BLA to the mPFC and HPC for an iterative loop that updates on the basis of experience. Furthermore, given that the MDT–mPFC circuit is important for cognition^143^, this pathway could serve to facilitate the cognitive and social-behavioural changes associated with social-rank learning. Given that social rank is dynamic and can change depending on the social context, the social rank of the individual and the other could provide a context-dependent factor to modulate social valence assignment. In addition, an individual’s internal state (for example, their hunger or isolation level) could change how social rank influences valence assignment of a conspecific.

## Social memory and valence assignment

Behavioural evidence supports the idea that social history affects interactions with a conspecific and the valence assigned to that conspecific. Humans and animals interact differently with strangers versus familiar conspecifics^144–148^. However, the neural dynamics and circuits underlying social history-related changes are unknown. Although social history is a broad term, several factors can be easily controlled and measured: familiarity, group size and social ranks of the individuals interacting. This parameterization enables social history to be studied in the laboratory setting. Unfortunately, in almost all studies of the neural circuits of sociability and social motivation to date, participants interacted with a novel conspecific, and neither group size nor social ranks were addressed as variables. Whether social-motivation circuits differ depending on social history is still an open question. Given the rich literature, we focus on the neural circuits for social recognition and social memory, and how they overlap with valence systems.

### Hippocampal circuits for recognition.

How does the brain recognize someone? The HPC is crucial for recognizing others and in forming and maintaining social memories. Individuals with hippocampal lesions are unable to recognize familiar faces or other familiar objects^149^. In the rodent social-recognition or social-discrimination test^5,8,150^, a mouse explores a chamber with two mice — a familiar and a novel conspecific — and the familiar mouse can be first encountered minutes or the day before testing to probe short-term or long-term social memory, respectively^6^.

Several studies have dissected the intrahippocampal circuits needed for social memory. The HPC contains subregions called the dentate gyrus and CA1, CA2 and CA3, which in turn have dorsal and ventral subdivisions^151^. Various hippocampal subregions play a part in social-memory encoding and the retrieval of social memories. For example, in rodents, the lateral entorhinal cortical projection to the dorsal dentate gyrus is necessary for the retrieval of short-term social memories^152^, and lesioning or ablating the dorsal CA2 disrupts social recognition but not sociability or other spatial-memory functions^153,154^. Furthermore, a study involving optogenetic and chemogenetic manipulation of the dorsal CA2 demonstrated that this region has a role in encoding, consolidation and retrieval of social memories^7^.

The neuropeptides oxytocin and vasopressin act in the HPC to facilitate social memory. Oxytocin receptors (OXTRs) are prominently expressed in the HPC^155,156^. Deletion of *Oxtr* in the rodent CA2 and CA3 disrupts 7-day-old, but not 1-day-old, social memory, and application of an *Oxtr* agonist facilitates potentiation in dorsal CA2 pyramidal neurons ex vivo^157^. However, another study showed that OXTRs in the dorsal dentate gyrus, CA2 and CA3 are necessary for short-term social recognition in the order of minutes^155^. Furthermore, input to neurons in the dorsal CA2 that express vasopressin receptor 1B from vasopressin-positive neurons in the paraventricular hypothalamus is necessary for encoding, but not for retrieving, social memories^158^.

The vHPC and several of its inputs and outputs also have a role in social memory and recognition. The projection from the dorsal CA2 and CA3 to the posterior CA1 is necessary for the retrieval of short-term social memories^155^, and the projection from the dorsal CA2 to the ventral CA1 is similarly necessary for the formation of short-term social memory^7^. Silencing either the ventral CA3 (REF.^159^) or the ventral CA1 (REF.^141^) disrupts social-memory recall. The number of ventral CA1 cells that encode a conspecific increases over 3 days of co-housing^141^, suggesting that social memory engrams in the ventral CA1 reflect familiarity level. Ventral CA1 projections to the NAc^141^ and to the mPFC^160^ are both necessary for short-term social memory. Furthermore, neurons in the dorsal CA2 target neurons in part of the ventral CA1 that project to the NAc, providing a multisynapse circuit that mediates social memory^7^.

By tagging activated cells^161,162^, a recent study shows that the vHPC contains mostly separable subpopulations of cells that encode either negative or positive valence^163^. However, these subpopulations are not anatomically divergent, as both vHPC–BLA and vHPC–NAc neurons routed negative and positive valence. By contrast, similarly flexible valence routing was not seen in the vHPC–mPFC pathway^163^. Collectively, these studies enable speculation about how valence signals in the vHPC might be integrated with social-memory information to control behaviour. Given that the vHPC–NAc pathway is necessary for social memory^141^ and encodes both positive-valence and negative-valence information^163^, it could induce social aversion or social preference depending on the context, whereas the vHPC–mPFC pathway routes valence-independent social identity signals, such as social rank.

### Other circuits for social memory.

Beyond the HPC, other circuits are also involved in social memory — particularly mPFC–NAc and mPFC–amygdala circuits. In rodents, neuropeptide signalling in the MeA is central to social-memory processes^8,164,165^ and encoding in the MeA changes with social experience, such as sexual experience^93–95^ (reviewed elsewhere^95^).

In addition, several prefrontal top-down circuits show a role in social memory — particularly those connecting to subcortical regions that have a well-established role in valence encoding (NAc and BLA). As social cues become familiar, the responses of mPFC cells to them decrease^97^, suggesting that the mPFC signals familiarity to guide social behaviour. Furthermore, projections from the infralimbic and prelimbic subdivisions of the mPFC to the NAc are implicated in social-memory processes. Inhibition of prelimbic neurons active during social interaction with a novel animal impairs social recognition, but not social preference^166^, implicating these neurons in social memory. These neurons are more likely to express D1 dopamine receptors than D2 receptors, suggesting their activity might be modulated by dopamine. Furthermore, inhibition of prelimbic NAc-projecting neurons disrupted recall of short-term social memory^166^. Consistent with this, prelimbic NAc-projecting neurons encode a combination of social and spatial information and have a role in spatial–social memory^117^. Another study showed that infralimbic neurons projecting to the shell of the NAc were more activated during exposure to familiar mice than novel mice and that this pathway was necessary for long-term social-memory recall^167^. Together, these studies implicate pathways from both mPFC subdivisions to the NAc in social memory. Finally, stimulating OXTR-expressing mPFC neurons projecting to the BLA impaired short-term social-memory recall, but not social preference or anxiety-like behaviour^168^.

Whether valence assignment of the social agent affects the circuits underlying social memory is unknown. Rodents can detect pheromonal signals of dominance in strangers^169–171^, and a recent study directly compared neuronal activation (assessed through immediate early gene expression) evoked by urine from familiar or unfamiliar dominant and subordinate conspecifics^116^. The neural responses of many brain regions, including the mPFC and amygdala, were modulated by a combination of familiarity and the social ranks of the test mouse and conspecific, implying that social history can modulate how the brain processes social cues. We hypothesize that, given the overlapping circuitry of social memory and valence processing, overlapping subpopulations of cells encode valence and social memory to support social memories with valence-specific information. Social memory and valence systems, together, can support valence assignment for familiar individuals based on social rank and context, by recalling memories of experiences with that individual in a given context.

Notably, animals can interact with the same individuals for long periods, and social valence assignment can change across time; therefore, there is a need to study longer-term social memories. Future work may determine whether the neural circuits and dynamics underlying long-term familiarity are the same as those underlying social memory over shorter-term periods of minutes or a day, and whether social rank of a conspecific affects the neural circuits underlying social memory. Moreover, the social attributes of familiar animals used in experiments should be examined as a variable in future work.

## Impacts of social isolation

**Fig. 2 |:**
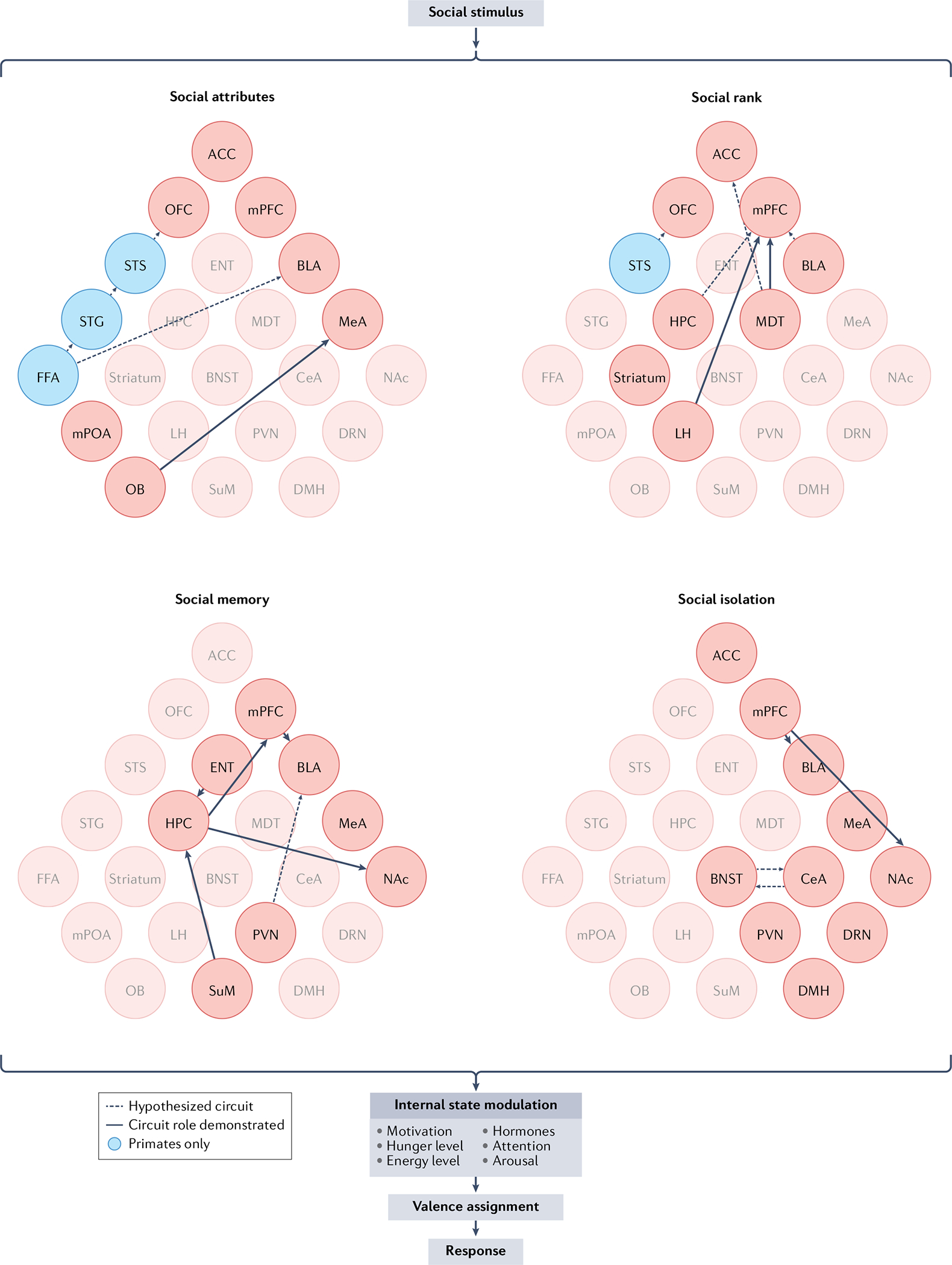
Factors influencing social valence assignment and circuits underlying those factors. A circuit summary of brain regions implicated in processing four factors that contribute to social context and, thus, assignment of social valence: social attributes, social rank, social memory and social isolation. Circuit nodes represented have been directly evidenced or hypothesized to play a part in processing these four social factors. Our central theory is that an animal assigns valence to a social stimulus depending on the attributes, rank, social memory and housing conditions of the individuals interacting. In addition, internal state factors such as hormones, energy and motivation affect valence assignment. Valence assignment, in turn, influences an animal’s behavioural response. Attribute circuits are based on primate^55,59,62,83,99,100,102,258^ and rodent^93–97^ literature. Social-rank circuits are based on primate^118,119,121–123,127,138,259^ and rodent^115,116,125,140^ literature. Social memory^117,141,152,155,160,168,260^ and social isolation^218,219,225,261,262^ circuits are based on rodent literature. Brain regions are not arranged anatomically, and regions found only in primate brain are coloured blue. ACC, anterior cingulate cortex; BLA, basolateral amygdala; BNST, bed nucleus of the stria terminalis; CeA, central amygdala; DMH, dorsomedial hypothalamus; DRN, dorsal raphe nucleus; ENT, entorhinal cortex; FFA, fusiform face area; HPC, hippocampus; LH, lateral hypothalamus; MDT, mediodorsal thalamus; MeA, medial amygdala; mPFC, medial prefrontal cortex; mPOA, medial preoptic area; NAc, nucleus accumbens; OB, olfactory bulb; OFC, orbitofrontal cortex; PVN, paraventricular nucleus of the hypothalamus; STG, superior temporal gyrus; STS, superior temporal sulcus; SuM, supramammillary nucleus.

**Fig. 3 |:**
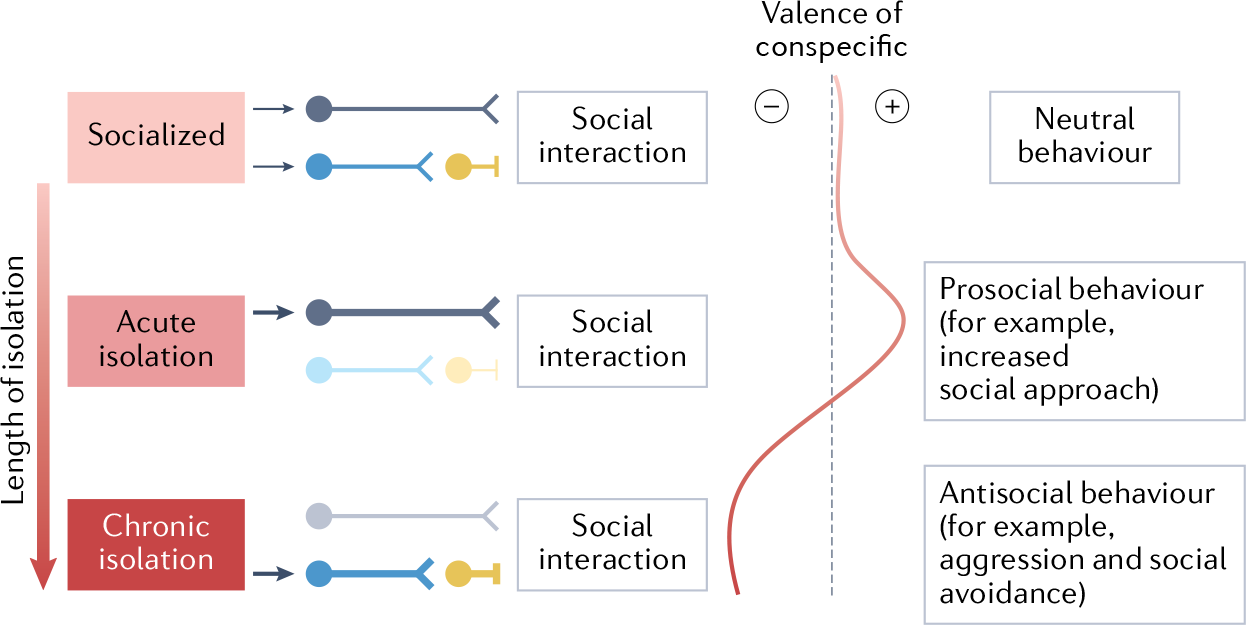
A model of the temporal effects of social isolation. As social isolation increases, prosocial behaviour first increases and then decreases as the duration of isolation extends, whereas antisocial behaviours increase. We hypothesize that the valence of a social conspecific transitions from neutral to positive to negative as an animal transitions from group housing to acute and then chronic isolation. A simplified account of how this could be achieved is depicted, whereby circuits from brain regions or molecules involved in encoding the internal states produced by isolation allow for the increase (acute isolation) or decrease (chronic isolation) in social interaction, by promoting or inhibiting activity in downstream social-motivation centres.

Following acute periods of social isolation, various animals — including rodents and humans — perform prosocial behaviours such as rebound social interaction and increased affiliative behaviours^3,32,33,172,173^. However, with chronic social isolation, flies, rodents and humans display antisocial behaviours, such as aggression, avoidance and social anxiety^25,26,174^, that may, in humans, manifest in the form of mental health disorders. These sequelae present an intriguing paradox — namely, how does the same experience of social isolation result in opposing effects on behaviour simply based on the duration of the experience (FIGS. 2 and 3)? How can the shift from prosocial behaviour associated with brief isolation to the antisocial behaviour associated with prolonged isolation be explained (FIG. 3)? Is this shift in behaviour the product of a change in the valence of social conspecifics from positive to negative as the period of isolation extends? When do deficits in social contact no longer drive prosocial behaviour? What adaptations occur when the frequency of opportunities for social engagement changes in a long-lasting manner? Recent advances have begun to shed light on how the brain encodes, or adapts in the face of, social isolation of various durations. Below, we discuss recent literature on the neural circuits, mechanisms and signalling molecules associated with the housing condition of an animal, highlighting studies focused on acute and chronic isolation and their impact on social valence.

### Acute social isolation.

The past decade has seen a considerable increase in understanding how the brain and behaviour are altered by housing conditions such as social isolation. Unfortunately, the notions of ‘acute’ versus ‘prolonged’ isolation are often relative, with no standard agreement for definitions in terms of lengths of time. Presumably, a distinction between these could be ascertained for each species or experimental backdrop, by determining the point at which isolation leads to a shift from performing prosocial behaviour to antisocial behaviour. However, isolation studies are often limited by a focus on either acute or chronic isolation, making this distinction unfeasible. Thus, here, we refer to periods shorter than 1 week as ‘acute’ and those longer than 2 weeks as chronic. These temporal cut-offs were selected based on the behavioural and neurobiological effects of each, with the behavioural effects of social isolation remaining similar from 1 h to 1 week, and the effects of chronic isolation emerging after 2 weeks of social isolation minimally and worsening with more time in isolation^26^. We focus on the effects of isolation in model systems, as investigations into the effects of isolation in humans has been well discussed in previous reviews^175,176^.

Social isolation has long been associated with negative effects on the brain and body^12,13,20,175,177–179^. However, more recent research has demonstrated the positive, prosocial effects of brief periods of social isolation^3,4,32,33,180–183^. Indeed, we previously found that 24 h of social deprivation resulted in mice showing an increase in motivation to seek social interaction with a novel conspecific, or ‘rebound sociability’^32^. This rebound sociability required midbrain dopamine neurons in the dorsal raphe nucleus (DRN), and correlated with an increase in activity of these neurons, supporting the intriguing idea that rebound sociability after isolation engages a DRN-specific dopaminergic pathway. Consistent with this, functional MRI blood oxygen level-dependent responses to social stimuli in the midbrain of humans were greater after 10 h of social isolation than before isolation^33^.

An overall role for dopamine in controlling social reward is well supported by the literature, as dopamine signalling by the ventral tegmental area has been implicated in the control of social interaction and reward under standard (group) housing conditions^184–186^. The sites of dopamine action to exert the effects of rebound sociability remain unknown, but dopaminergic neurons in the DRN project to various regions implicated in social and emotional regulation (see below). Notably, D1 and D2 receptors in the NAc are required for acute isolation to promote social interaction in rats^187^. Thus, region-specific dopamine signalling might mediate prosocial behaviour depending on environmental conditions, although the role of such signalling during the shift from acute to chronic isolation has not been examined.

Interestingly, the potentially rewarding effects of rebound sociability seem to interact with social status: low-ranking animals exhibit less rebound sociability and DRN dopamine activity than do high-ranking animals^32^. Thus, the value of (even a brief) social interaction strongly depends on an animal’s social standing, revealing the bidirectional relationship between housing condition and social rank in determining the rewarding value of a conspecific.

Beyond dopamine changes, acute social isolation also induces various physiological and behavioural changes, including altered immune responses, changes to hypothalamus–pituitary–adrenal (HPA) axis activation, and heightened arousal and defensive behaviours (reviewed elsewhere^4^). These changes signal an acute state of social withdrawal and may set the stage for increased responding to subsequent social stimuli.

Finally, acute social isolation is associated with changes in various signalling molecules across many brain regions (reviewed elsewhere^3^). Briefly, acute isolation has been shown to produce both increases or decreases in the expression of corticotropin-releasing hormone (CRH) and/or CRH receptor depending on the brain region interrogated^188,189^, as well as decreased excitability of CRF-expressing neurons in the paraventricular nucleus of the hypothalamus (PVN)^190^. Acute isolation also produces a decrease in mPFC GABA levels^191^. Oxytocin, known for its role in pair bonding^192^, is also implicated in social isolation. For example, chemogenetic inhibition of oxytocinergic neurons in the PVN reverses the effects of acute isolation on social interaction^193^. Also, chronic systemic delivery of oxytocin blocks the effects of prolonged isolation on subsequent antisocial behaviour^194,195^. These data highlight the multiregional, multi-mechanistic and multi-neurochemical way in which brief periods of social isolation can affect prosocial behaviour.

### Chronic social isolation.

Unlike acute isolation, prolonged periods of social isolation produce deleterious effects on behaviour, including increased avoidance of social conspecifics and increased antisocial behaviours. In humans, loneliness — the internal state of perceived social isolation — is associated with depression, irritability and increased mortality^176^. Solitary confinement, the most extreme form of social deprivation, is linked to poor mental health outcomes, aggression and loss of emotional control^196^.

For decades, the study of chronic social isolation in primates had been deemed unethical following the notorious studies of maternal separation in rhesus monkeys by Harlow and colleagues showing that infant monkeys preferred the warm comfort of a soft mother-like structure over a wire mother that supplied milk, suggesting that the soft touch, or ‘contact comfort’, is more important than a food source^197,198^. These studies resulted in long-lasting and largely irreversible negative consequences on the maternally deprived infants^197,198^. As a result, research on early-life stress, including maternal separation, was largely relegated to rodent models^199–204^, and support for research into social isolation was substantially reduced.

Now, in light of a pandemic that has precipitated an unprecedented level of social isolation, social distancing and social exclusion, the prominent omission in our understanding of the neurobiological consequences of reduced social contact is glaring. Poignantly, the pandemic has produced an increase in violence as well as depression and anxiety, hypothesized to constitute a ‘second pandemic’ of social isolation^205–207^.

Chronic social isolation generates an increase in antisocial behaviour in various species. Long-term isolation has long been used in fruitflies, mice, rats and other species to increase aggressivity towards a conspecific^28,29,208–212^ in males and females^213–215^. In addition, chronic social isolation reduces social investigation and motivation to interact with a conspecific^26^. Importantly, in mice, acute isolation had no effect on aggression or alterations in fear, in contrast to chronic isolation, which increases aggressivity and persistent fear responses^26^. In the same study, chronic social isolation was shown to increase threat responsivity, fear-related behaviours and risk-taking behaviours, and to reduce time spent in a chamber containing a novel conspecific in the three-chamber assay. These results contrast with findings that acutely isolated mice spend more time interacting with a conspecific in the same assay^32^, and that other species similarly show an increase in social interaction after acute isolation^172,216,217^. These results suggest that prolonged social isolation produces a unique, deleterious internal state.

Recently, there has been considerable progress in our understanding of the neural circuitry and molecular mechanisms that underlie the effects of prolonged social isolation. For example, one study of the effects of chronic social isolation in juvenile mice^218^ showed that mPFC neurons have a dissociable role in isolation-induced aggression in males and isolation-induced reductions in sociability in females. These behavioural changes correlate with changes in the spiking activity of cells in regions downstream of the mPFC — including the BLA and ventral tegmental area in males and females, respectively. These studies provide further support for the role of mPFC in providing top-down control of aggression^219^.

We recently implicated subcortical structures in the brain state produced by prolonged social isolation. Multiplexed loss-of-function approaches revealed dissociable roles for the neuropeptide tachykinin 2 (TAC2) in the anterior dorsal bed nucleus of the stria terminalis (BNST), dorsomedial hypothalamus (DMH) and central amygdala (CeA) in the control of isolation-induced persistent fear, enhanced aggression and acute fear, respectively^26^. In addition, brain-wide overexpression of TAC2 combined with chemogenetic activation of TAC2-expressing neurons induced behaviours in group-housed mice that mimicked those of isolated mice, including increased fighting and persistent fear responses, and these effects could be reversed by a TAC2-receptor antagonist^26^. Therefore, TAC2 signalling is necessary and sufficient to induce a social isolation-like state. Although neuromodulators and neuropeptidergic subpopulations have been implicated in the regulation of internal states and certain behaviours, respectively^220–225^, this study describes how a single neuropeptide system acts in different brain regions in concert to mediate the internal state produced by chronic social isolation and exert control over isolation-induced behaviours.

Many studies also suggest a role for glucocorticoids and the HPA axis in regulation of the effects of prolonged social isolation (reviewed elsewhere^179^). Similar to other stressors, social isolation increases cortisol levels^176,226^. Intriguingly, whereas acute social isolation results in various changes in the expression of HPA-related genes, these changes usually dissipate as the isolation period grows, suggesting that the HPA axis soon adapts to counteract the effects of isolation^179^. By contrast, following chronic periods of social isolation, HPA axis dysregulation is more likely to persist^179^.

### An integrated social homeostasis model.

We believe that social isolation is unique among stressors in its ability to have opposing effects on social behaviour depending on its duration. Indeed, opposing effects of acute versus chronic social isolation on feeding and sleep have also been identified, whereby chronic, but not acute, social isolation increases feeding behaviour and reduces sleep in *Drosophila*^227^. Other stressors produce effects that may vary with the stressor’s intensity, duration or proximity, but they tend to maintain the same negative effects and valence^228–234^. What is the evolutionary benefit of the contrasting effects of short-term and long-term isolation?

We have previously hypothesized that, similar to hunger, isolation engages a homeostatic mechanism to appropriately control an animal’s response to a social conspecific depending on housing conditions^3,4^. Thus, following acute social isolation, the detector would send information to the control centre, which would compute a deficit relative to the homeostatic set point and, thus, activate effector systems that would drive the animal to emit behaviours that would increase social contact to bring the detected level of social contact back to the homeostatic set point^1,35^. Indeed, the idea that low levels of stress are biologically beneficial has long been supported, consistent with the prosocial behavioural response following acute isolation^33,172^.

How can the same condition (isolation) and the same stimulus (social group) shift from positive to negative valence? As described above, short periods of acute social isolation produce prosocial behaviour, which switches to antisocial behaviour as the period of isolation increases. Thus, we hypothesize that the valence of the same social stimuli would shift from positive to negative depending on whether there is a perceived deficit or surplus of social contact detected relative to the social homeostatic set point, respectively (FIG. 3). With acute isolation, the effector system is activated, increasing correction effort (energy/time/resource expenditure towards obtaining the homeostatic set point, the preferred optimal). When an acutely isolated individual is reintroduced to the social group, a rebound of social interaction and affiliative behaviour may serve to restore the detected social contact to the homeostatic set point. By contrast, prolonged isolation may eventually trigger the recalibration of the social contact optimum (known as ‘set point adaptation’)^4^, such that the previous optimum level of social contact may now be perceived as a surplus. According to this hypothesis, chronically overcrowding animals would also cause a resetting of social homeostasis that would increase future basal preferences for social contact. These models provide a theoretical framework by which the divergent effects of acute and chronic stress can be reconciled^4^.

It should be noted that this hypothesis suggests that all antisocial behaviours are elicited by or directed towards a social stimulus with a negative valence. Such a hypothesis would seem to contrast with the finding that aggression is rewarding, such that rodents will perform operant behaviours to receive access to a conspecific whom it can attack^235^. Importantly, however, such studies often require that the conspecific mouse is more docile (often by having previously and repeatedly been defeated) and that the aggressor mouse is prescreened for increased aggression-seeking behaviour or has been previously exposed to fighting experiences in which it ‘won’. In contrast to these set-ups, social isolation-induced aggression may occur with no prior aggression training or knowledge of the potential ‘outcome’ of an upcoming social interaction^236^. This argues against the idea that opponents during attack are attributed with a positive valence. Furthermore, although an animal might seek an aggressive encounter, this does not necessarily distinguish between the rewarding properties of ‘winning’ and the rewarding nature of fighting. Additional studies that manipulate variables related to conspecific valence and reward are warranted to further isolate the mechanisms controlling social valence during acute versus prolonged social isolation.

## Conclusions and looking forward

In this Review, we have defined social valence, reviewed the rich literature in this growing field enabled by new technologies^237–240^ and put forth a simple conceptual framework outlining testable hypotheses to probe the key parameters in socio-emotional processes. For example, when animals are in competition, they represent outcomes for the self and the other separately^125,132^. We hypothesize that neurons in non-overlapping ensembles^241^ may help represent the other’s outcome as either aligned (overlapping) or orthogonal (non-overlapping) depending on whether or not the animals are in cooperation (aligned representations for self and other) or in competition (orthogonal representations for self and other)^125^. More empirical data will be needed to inform construction of a quantitative, predictive model for how both internal inputs (for example, relating to hunger, social memory and emotion state) and external inputs (for instance, resource scarcity, dominance behaviours exhibited by conspecifics and competitive success) are integrated and weighted to ultimately determine behaviours from the individual and other social agents.

But how is social valence constructed, and what information is integrated to abstract down to the general property of valence? Despite our knowledge of the individual factors that influence social valence and their individual neural systems, we do not know how they interact with each other. Future experiments could test the hypothesis that they have a hierarchical nature, with some factors, such as the isolation state, being higher in the hierarchy and weighted more for social valence assignment than other factors. Alternatively, attributes that contribute to social recognition and valence identification from features (such as faces, voices and so on) could have the largest role. How social attributes and familiarity interact is unknown, and future experiments could address how social history and memories affect social attribute perception. Partially overlapping neural circuits are involved in the perception of social attributes, social rank and history, and many of these contain subpopulations of neurons that encode valence (including those in the mPFC, ACC, BLA, HPC and NAc), highlighting a potential neural mechanism for social valence assignment.

As a field, the study of the neural mechanisms of social hierarchies is still in its infancy but animal models and evidence of both innate and learned mechanisms to support hierarchies and social-rank encoding are already proliferating^9^. New studies that take into account previous history of group composition and size will be crucial to understand how history affects rank development and hierarchical placing, whereas other studies in more controlled environments can dissect how the mechanisms of reward and aversion encoding affect social hierarchy formation. Work across species suggests differences in the dopaminergic and serotonergic systems in social dominance^242–246^. Considering the role of neuromodulation in valence assignment^133,247,248^, future work may focus on understanding how neuromodulatory systems contribute to rank-dependent social valence assignment.

Interestingly, social isolation itself seems to be a powerful modulator of social valence. We speculate that isolation is a powerful modulator because of evolutionary drives and the importance of socializing for reproduction and defence. Furthermore, we have theorized that the state of social isolation influences the social valence of other social factors. In this model, chronic isolation alters how attributes, history and rank are perceived. Few studies have looked at how isolation state affects other social factors and their neural circuits and dynamics. For example, is the increase in aggression seen after social isolation as rewarding as aggression seen during territorial defence? Which factors can explain the effects of prolonged isolation on reducing approach behaviour but also increasing aggression simultaneously? Can we uncouple the valence assigned to a social conspecific from the valence assigned to the social interaction (such as a fighting episode) experienced with that same conspecific? Also, what are the circuit or neurochemical mechanisms that explain an animal’s shift in behaviour from acute to prolonged social isolation? Is this shift more or less dramatic depending on an animal’s position in its social hierarchy? At least one study indicates that social rank can affect the consequences of social isolation^32^, suggesting that social factors can interact with the isolation state to affect social valence assignment. Are there ways to protect against the deleterious effects of prolonged isolation? What role does control, or the perception of control, have in the development of these negative effects? Future studies aimed at teasing apart these factors will shed light on the neurobiology underlying social isolation and its impact on behaviour.

Collectively, understanding how social perception, contextual factors, prior history and isolation state are integrated in the brain to control social motivation is vital, given the high prevalence of dysregulated social behaviour in psychiatric and neurological disorders^249^.

## Box 1 | Social valence is guided by the alignment of goals for self and other

It is adaptive for individuals of a species that share genetic material to cooperate and to care for others as extensions of themselves. By contrast, it may be more adaptive for a species overall if only a subset of genetic material (that of the ‘fittest’) is perpetuated2^263^, thereby necessitating a competitive scenario both within and between species (FIG. 1). Interestingly, this evolutionary theory has been supported by behavioural observations showing that rats show more altruistic ‘helping’ behaviour when presented with familiar rats or rats sharing physical traits of those with which they were raised^264,265^. Individuals do not seem to be inherently aware of their own genotype but, instead, rely on familiarity and stimulus generalization for identification, as evidenced by the fact that rats will more readily help a stranger that is similar to those they were reared with, even if they are a different strain^264^. This notion is also supported in humans by racial and gender biases that are pervasive (as reflected in Harvard’s Implicit Association Test^266^) and influenced by the racial identity of the participant, and by the finding that interracial cohabitation promoted subjective pleasure and physical engagement while reducing anxiety in a novel interracial interaction^267,268^.

Some work has identified possible neural mechanisms that help an individual determine whether their own goals are aligned with those of a conspecific. Brain state synchrony of interacting individuals in the prefrontal cortex (PFC) has been shown in humans^269^ and mice^270^ alike. In the context of social decision-making during a prisoner’s dilemma task, which requires animals to anticipate the social alignment of others, non-human primates interacting or engaging in cooperative behaviours show distinct activity in corticolimbic circuits involving cortical regions such as the anterior cingulate cortex (ACC), PFC, orbitofrontal cortex (OFC) and basolateral amygdala (BLA) from that shown during competition^132,253,255,271,272^. Furthermore, a recent study in macaques shows that activity in the dorsal medial prefrontal cortex (mPFC) encodes detailed representations of a group’s behaviour and reflects others’ identities, actions and outcomes^273^.

## Box 2 | Aggression, altruism, empathy and observational learning

Here, we introduce the social behaviours and psychological constructs of aggression, altruism, empathy and observational learning, and explore some of the neural mechanisms thought to mediate them.

In mice, circuits for aggression — largely identified as those mediating increases in biting bouts and overall time spent attacking (including biting, tussling and lunging) — have been identified in mice in the ventromedial hypothalamus3^215,235,274,275^, pinpointing neuronal ensembles that orchestrate complex social behaviours in a dynamic and experience-dependent manner^276^. Recent work extends the sensitivity of the ventromedial hypothalamus to social experience and implicates this region in the encoding of additional social experiences (such as social defeat), and the representation of environmental cues that predict these negative experiences^277^. Circuits involving the nucleus accumbens (NAc) in the ventral striatum that encode the rewarding aspects of aggression on others have been identified using a task in which mice perform an operant response to gain access to a conspecific over which they will exert their aggression^278^, with relevance to sadism and bullying. Furthermore, in primates, striatal neurons that encode reward also encode the social agent when there are two monkeys involved in the task^279^.

The notion of empathy, the ability to recognize the emotions of others and to take on that emotion^280^, is distinct from the related concept of altruism^281^ (although it has been conceptualized as correlated^281^), which focuses on helping another at one’s own expense. Altruism may rely on the ability to recognize distress in others, but does not necessarily require the taking on of the emotions of others^282^, as occurs in empathy. Studies in humans have identified the amygdala, insular cortex, orbitofrontal cortex (OFC) and anterior cingulate cortex (ACC) as key regions involved in empathy^283^, and mirror neurons are also theorized to facilitate empathy^284^.

Another important distinction is that between the related concepts of empathy and observational learning. In observational learning, an individual can learn about the valence of environmental stimuli, as well as appropriate responses to them, by observing another individual, without direct experience^285^. Observational learning can be applied to both positive and negative valence, providing an enormous adaptive advantage. For example, it could be costly or fatal to learn through direct experience that a predator is a threat; thus, learning that a predator is threatening by observing another animal’s escape responses or defensive behaviours in response to a predator-associated cue enables learning without the same cost. During observational learning, the ACC detects and represents information about the behavioural state of the social agent and routes this information to the basolateral amygdala (BLA), where ACC input is crucial for observational learning^286^. The related psychological constructs of empathy, cooperative and competitive decision-making and observational learning all implicate largely overlapping cortical circuits, including the ACC and BLA ensembles^287–289^, suggesting that the biological implementation of these related computations is also related.

## Abbreviations

Sociability: A parameter describing the degree to which an animal seeks social contact or engages in social interactions.
Valence: The degree to which something is pleasurable (positive) or aversive (negative).
Social context: The social aspects related to the environment that an individual is in. This can include social rank and the presence or absence of others, as well any history of social interactions (such as fighting, mating and so on) in that context.
Social rank: The social position of an individual relative to others in a group (for example, social hierarchy).
Social valence: The valence assigned to a social stimulus or agent.
Cooperation: Two or more individuals with common or shared goals.
Competition: Two or more individuals with goals which are incompatible or in conflict.
Social alignment: The degree to which the goals of the self are in cooperation (‘aligned’) versus in competition (‘opposed’) with the goals of the other.
Social history: The collective social experience of an individual. This includes social memories, social rank, isolation history, group size and other related social experiences.
Social attributes: The physical attributes of a social agent including their age, size, rank, resource-holding potential and so on.
Social heuristics: Generalized associations that can facilitate rapid assessment of a social agent.
Brain state synchrony: When the internal brain state of a social agent is synchronized to the brain state of another social agent, such that changes in one produce changes in the other.
Theory of mind: The ability to create a model of another’s mind as distinct from one’s own by inferring their mental state, logic, beliefs and emotions.
Emotional contagion: The phenomenon of individuals mimicking the emotions or emotional behaviours of others.
Social homeostasis model: A conceptual model proposing that there is an optimal quality and/or quantity of social contact, regulated by a detector, control centre and effector system.
Social dominance: The repertoire of behaviours expressed by higher ranking animals including winning during competition and species-specific body poses.
Mixed selectivity: The ability of neurons to respond consistently to multiple, statistically independent variables.
Valence associative learning: Learning the association between two or more stimuli in which one of the stimuli has a positive or negative valence.
Familiarity: The degree to which two individuals know each other.
Engrams: Ensembles of neurons that undergo enduring changes during learning and facilitate memory recall.
Social preference: The preference for social stimuli (as opposed to non-social stimuli) observed in rodents.
Spatial–social memory: Memory of the space in which a social interaction occurred, often indexed by changes in the time spent in that social space.
Rebound social interaction: The increase in social interaction observed immediately after a social deficit.
Social anxiety: The decrease in social interaction and/or social preference observed following a negative social experience (for example, prolonged isolation, social defeat and so on).
Threat responsivity: The degree to which a host of behavioural responses are expressed in response to a threatening, noxious stimulus, including: freezing, darting, activity bursting, flinching, vocalizations and so on.
Homeostatic set point: Within any homeostatic system, there is a control centre that stores a ‘set point’ and computes the difference between the detected input and the optimal set point.
